# Injection drug use and patterns of highly active antiretroviral therapy use: an analysis of ALIVE, WIHS, and MACS cohorts

**DOI:** 10.1186/1742-6405-4-12

**Published:** 2007-06-06

**Authors:** John D Morris, Elizabeth T Golub, Shruti H Mehta, Lisa P Jacobson, Stephen J Gange

**Affiliations:** 1 Department of Epidemiology, Johns Hopkins Bloomberg School of Public Health, Baltimore, Maryland, USA

## Abstract

**Background:**

Sustained use of antiretroviral therapy has been consistently shown to be one of the primary predictors of long-term effectiveness. Switching and discontinuation reflect patient and provider decisions that may limit future treatment options. In this study, we utilize data reported at semi-annual study visits from three prospective cohort studies, the AIDS Link to IntraVenous Exposure (ALIVE), the Women's Interagency HIV Study (WIHS), and the Multicenter AIDS Cohort Study (MACS), to investigate determinants of HAART modification with a particular focus on reported injection drug use (IDU).

**Methods:**

Longitudinal data collected between 1996 and 2004 contributed from 2,266 participants (37% with a reported history of IDU) who reported initiating their first HAART regimen during follow-up were utilized. Separate proportional-hazards models were used to identify factors measured prior to HAART-initiation associated with the time to first HAART discontinuation and first switch of components of HAART among continuous HAART users.

**Results:**

The use of PI- vs. NNRTI-based regimens among HAART users with and without any history of IDU was similar over follow-up. The median time to a first report of discontinuation of HAART was 1.1 years for individuals with a history of IDU but 2.5 years for those without a history of IDU and multivariate analyses confirmed overall that individuals with a history of IDU were at greater risk for HAART discontinuation (adj RH = 1.24, 95% CI: 1.03–1.48). However, when restricting to data contributed after 1999, there was no longer any significant increased risk (adj RH = 1.05, 95% CI: 0.81–1.36). After adjusting for pre-HAART health status and prior ARV exposure, individuals who were ethnic/racial minorities, reported an annual income < $10,000/year, and were not employed were at significantly greater risk for HAART discontinuation. The median time to a first change in HAART regimen was approximately 1.5 years after first HAART report and was not elevated among those with a history of IDU (adj RH = 1.09, 95% CI: 0.89–1.34).

**Conclusion:**

Our analyses demonstrate that injection drug use by itself does not appear to be an independent risk factor for HAART switching or discontinuation in more recent years. However, as continued HAART use is of paramount importance for long-term control of HIV infection, efforts to improve maintenance to therapy among disadvantaged and minority populations remain greatly needed.

## Background

Highly active antiretroviral therapy (HAART) has been unequivocally associated with improved survival among individuals infected with human immunodeficiency virus (HIV) and has decreased the incidence of AIDS-associated opportunistic infections [1,2]. However, as HIV-infected patients begin to live longer on HAART therapy, issues regarding viral resistance, short and long-term drug toxicities, and adherence due to complex regimens have become important concerns [3-5]. These issues, in addition to the increasing number of treatment options, make it often necessary to modify therapy in order to achieve the goals of viral suppression, longer survival, and improved quality of life [6]. These modifications in treatment, either switching to a new regimen consisting of a different drug or drugs, downshifting to a non-HAART treatment regimen, or discontinuation of antiretroviral use, have uncertain effects on the entire course of disease and may limit the number of future treatment options [7-10].

Several previously published studies have characterized modifications of HAART regimens by determining the time before HIV patients discontinue or switch to a new HAART regimen and have investigated correlates of therapy modification or discontinuation [8,10-17]. One factor that has not been well addressed is whether individuals with a history of injection drug use (IDU) are more likely to modify their HAART regimen. Injection drug use represents a major route of infection in the US and other parts of the world and presents particular challenges for HIV treatment, including "the existence of an array of complicating co-morbid conditions, limited access to HIV care, inadequate adherence to therapy, medication side effects and toxicities, need for substance abuse treatment, and the presence of treatment complicating drug interactions." [18] Previous studies suggest that a history of injection drug use may be a possible factor in determining the duration of HAART regimens [11,14] although few studies of therapy modification have included substantial numbers of individuals with a history of injection drug use.

The purpose of this study was to investigate the occurrence of HAART modifications among individuals enrolled in the AIDS Link to IntraVenous Experience (ALIVE) study, a prospective cohort study of individuals with a history of injection drug use in Baltimore, Maryland [19]. These data were combined with data from two other US prospective cohort studies that had a smaller proportion of individuals with a history of injection drug use: the Women's Interagency HIV study (WIHS) [20] and the Multicenter AIDS Cohort Study (MACS) [21]. We compared and contrasted the frequency of HAART modification among those with and without a history of IDU across diverse populations, hypothesizing that participants with a history of IDU would be more likely to switch or discontinue their initial HAART regimen. Our approach capitalized on commonality of data collection instruments and use of common variable definitions, selection criteria, and algorithms for determining HAART regimen changes.

## Methods

### Study design

The MACS, WIHS, and ALIVE studies are all prospective studies of HIV infection in the United States. The MACS is a multicenter study composed of 6,972 men who have sex with men: 4,954 were recruited in 1984, 668 were recruited in 1987–1991, and 1350 recruited in 2001–03 in Baltimore, Chicago, Los Angeles, and Pittsburgh. The WIHS is also a multicenter study, with 2,623 women recruited in 1994–95 and 1,143 recruited in 2001–02 from New York (2 sites, Bronx and Brooklyn), Chicago, Los Angeles, San Francisco, and Washington DC. The ALIVE study, located exclusively in Baltimore, is comprised of 3,360 participants with a history of drug injection, of whom 2,921 were recruited in 1988–89 and 439 were recruited in 1994. All three studies employed similar follow-up data collection methods in which participants returned semiannually for a physical examination and an interview-based questionnaire to obtain information on demographic and psychosocial factors as well as information regarding medical history including antiretroviral medication use. Participants were asked a series of detailed questions about each antiretroviral medication they had taken since their previous visit and whether they were taking that medication at the time of the study visit. Photo-medication study aids were used to facilitate the participants in recalling antiretroviral medication use. Blood specimens were collected for quantification of plasma HIV RNA viral load and CD4 cell counts at each visit using standardized techniques. Additional information regarding the study design, population, and demographics of each study has been reported previously [19-21].

### Definitions of therapy and treatment modification

A participant was considered to be on HAART therapy if the regimen they reported met one of the following criteria: 1) at least two nucleoside/nucleotide reverse transcriptase inhibitors (NRTIs) with at least one protease inhibitor (PI) or one non-nucleoside reverse transcriptase inhibitor (NNRTI), 2) one NRTI and at least one PI and at least one NNRTI, 3) an abacavir- or tenofovir-containing regimen with at least 3 NRTIs, 4) a regimen containing ritonavir and saquinavir and one NRTI, and no NNRTIs. Combinations of zidovudine and stavudine were not considered HAART because of their contraindication. This definition of HAART was in accordance with the guidelines for antiretroviral therapy established by the DHHS [18]. A participant was considered to be receiving combination therapy when they reported using two or more antiretrovirals but their regimen did not meet the above criteria for HAART. Monotherapy was considered to be the use of only one antiretroviral of any class.

HAART regimens were grouped into four types depending upon the class of antiretroviral drugs reported: 1) PI with no NNRTIs, 2) NNRTI with no PIs, 3) both PI and NNRTI (dual), or 4) PI and NNRTI sparing (neither a PI nor NNRTI – i.e. abacavir or tenofovir combinations as described above). This classification of HAART regimens was used to evaluate the trends of type of HAART use over time in the three cohorts.

Modification of HAART regimen was assessed by comparing consecutive semi-annual study visits. Changes from one visit to the next were classified in three ways [15]: 1) *HAART switching *occurred when a regimen was modified such that at least one drug was different in the regimen but the participant was still on HAART according to the above definitions; 2) *Downshifting *occurred when a participant who was previously on HAART reported using a less intense regimen that only met the definitions of either combo- or monotherapy; 3) *Discontinuation *occurred when a participant stopped using all antiretrovirals completely. Those HAART users who reported the same regimen at consecutive visits were considered stable HAART users. Participant visits where a combination of drugs were reported to be used since their last visit that met the definition of HAART but, at the time of the visit, a regimen that did not meet our definition of HAART was reported, were excluded because report of drugs used since the last visit were not necessarily used concomitantly. Therefore, this exclusion was implemented to ensure that only participants who were unquestionably on HAART regimens were being evaluated.

### Study sample and data analysis

A total of 269 HIV infected participants from ALIVE, 1,301 from WIHS, and 696 from MACS attended a study visit at least once between April 1, 1996 and April 1, 2004, reported initiation of HAART while under active follow-up prior to October 1, 2004 and had pre-HAART CD4+ count data available.

Our analytical methods aimed to accomplish two goals. First, we assessed longitudinal trends in the use of HAART by comparing the proportion of HAART use comprised of each regimen type, as described above. The impact of cohort (ALIVE, MACS, or WIHS) and injection drug use history on the prevalence of each of the regimen types (PI only, NNRTI only, dual PI/NNRTI, and PI/NNRTI sparing) was evaluated using logistic regression models adjusting for calendar year. We adjusted for the correlation among repeated measurements within a year using generalized estimating equation techniques.

Second, the prevalence of switching, downshifting and discontinuation over time was examined. To determine whether history of injection drug use was associated with HAART modifications, Kaplan-Meier methods and Cox proportional hazards models [23] were fit to determine the cumulative incidence and relative hazards of modifying the first HAART regimen. Analyses were conducted with two different outcomes and risk sets: (1) we used data from individuals with study visits after HAART initiation to evaluate the time from first reported use of HAART to the first time of HAART discontinuation or downshifting, and (2) we used data from individuals reporting consistent HAART use to evaluate the time from first reported use of HAART to the first time of switch of HAART regimen. For each model, the primary exposure variable of interest was history of injection drug use. Additional factors included in multivariate models were measured up to the time of HAART initiation: age at HAART initiation, race/ethnicity (black, Hispanic, or other), sex, nadir pre-HAART CD4+ lymphocyte count, peak pre-HAART HIV viral load, prior AIDS diagnosis, pre-HAART (baseline) antiretroviral experience, alcohol and tobacco use, employment, and income. In each of these models, we adjusted for overall secular trends using calendar time as a time-varying covariate. However, because the observed differences in HAART modification patterns appeared to change over the course of follow-up, we also report the results of a model limited to data contributed in 1999 or later (after abacavir had been approved and began to be reported in the cohorts). This included data from individuals who initiated HAART in 1999 as well as from individuals who initiated earlier – the latter contributions are considered left-truncated and contribute to the analysis using standard staggered (late) entry techniques.

Lastly, we also examined the association of time-varying report of injection drug use with separate analyses. First, we included a time-varying indicator of current IDU in the model that also controlled for history of injection drug use. Second, we refined the analysis to compare individuals reporting current and former injection drug use with those never reporting a history of injection drug use.

## Results

### Participant characteristics

Pre-HAART characteristics of the 843 participants with and 1,423 participants without a history of injection drug use are presented in Table 1. Of the 269 ALIVE participants, 100% had a history of injection drug use (by design), while only 37% (475/1,301) of WIHS participants and 14% (99/696) of MACS participants had such a history. Those without a history of injection drug use had statistically (p < 0.01) higher nadir CD4+ lymphocyte count, and were significantly less likely to be Black, non-Hispanic (and more likely to be White or Hispanic), had higher education and income, were more likely to be employed, were less likely to be current smokers or drinkers, less likely to have ever been diagnosed with AIDS, and initiated HAART earlier (pre-1998) than those with a history of injection drug use.

**Table 1:** Participants' characteristics at first HAART visit

	**No history of injection drug use**	**History of injection drug use**
**No. ever reporting HAART**	1,423	843
**Study (% male):**		
**ALIVE**	0 (NA)	269 (72%)
**MACS**	597 (100%)	99 (100%)
**WIHS**	826 (0%)	475 (0%)
Race/ethnicity:***		
**Black, non-hispanic**	35%	62%
**White, non-hispanic**	45%	23%
**Hispanic**	19%	14%
**Median (IQR) nadir CD4 count (cells/mm**^3^)***	187 (76–315)	152 (64–251)
**Median (IQR) peak plasma HIV RNA (cps/ml)**	90,606 (25,000–250,000)	84,000 (21,734–269,943)
High school education (vs less)***	74%	58%
Less than $10,000 legal income***	39%	72%
Currently employed***	47%	23%
Current smokers***	33%	73%
Current drinkers***	21%	28%
Ever prior AIDS diagnosis***	34%	45%
Year of HAART initiation***		
**pre-1999**	75%	62%
**1999–2001**	16%	31%
**post-2001**	9%	7%
**HAART class**		
**PI/No NNRTI**	61%	62%
**No PI/NNRTI**	18%	18%
**PI/NNRTI**	5%	5%
**No PI/No NNRTI**	16%	15%

***p < 0.001

### Patterns and trends of HAART use

**Figure 1:**
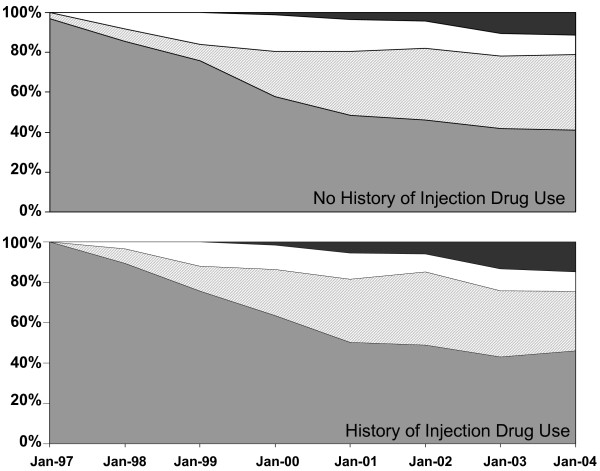
The proportion of HAART users taking a regimen containing 1) a PI but no NNRTI (dark gray), 2) a NNRTI but no PI (light gray), 3) both a PI and NNRTI (white), 4) neither a PI nor NNRTI (black), plotted over time for each study. The proportion is plotted at the midpoint of each visit window.

The longitudinal trends in the use of HAART by class of drug are depicted in Figure 1. Both individuals with and without a history of injection drug use showed a decreasing proportion of PI-based HAART over time, with a small proportion reporting dual PI/NNRTI and PI/NNRTI sparing regimens. Results from logistic regression analyses did not show any consistent significant differences between studies or between individuals with or without injection drug use. For example, the proportion of participants on HAART using a PI decreased from 98% in ALIVE, 94% in MACS, and 93% in WIHS in July 1997 to 51%, 56%, and 56% respectively in January 2004.

**Figure 2:**
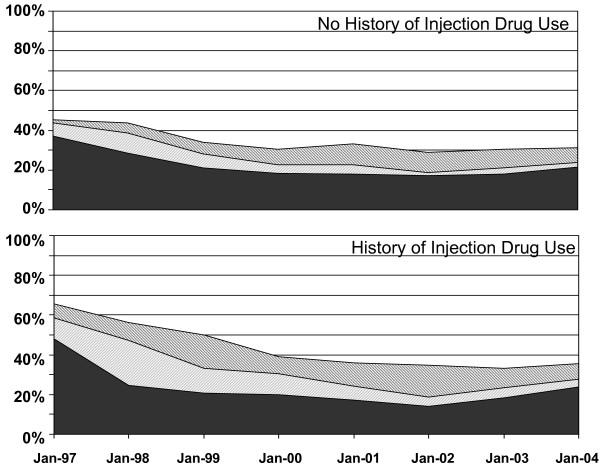
The prevalence of switching (black), downshifting (white), and discontinuation (gray) is plotted over time for each study at the midpoint of each visit window.

Figure 2 displays trends in HAART switching, downshifting and discontinuation between consecutive visits among those with and without a history of injection drug use. The proportion of participants on HAART who reported using the same HAART regimen at their next follow-up visit (stable HAART use) increased considerably over time in all three studies. Further, the proportion using the same HAART regimen was higher for those with no history of injection drug use. For example, the prevalence of stable HAART use in those with no history of injection drug use increased from 55% in 1997 to 70% by 2004. In contrast, the prevalence of stable HAART use in those with a history of injection drug use started lower, 35%, in 1997 and increased to 65% by 2004. There appeared to be larger differences between groups prior to 1999. Most modifications to HAART were HAART drug component switches (20% overall) followed by discontinuation (9% overall) and downshifting to monotherapy or non-HAART combinations (6% overall). Because of the relatively small numbers, the discontinuation and downshifting outcomes were combined into a single "discontinuation" outcome for subsequent analyses.

### Predictors of first HAART modification

**Figure 3:**
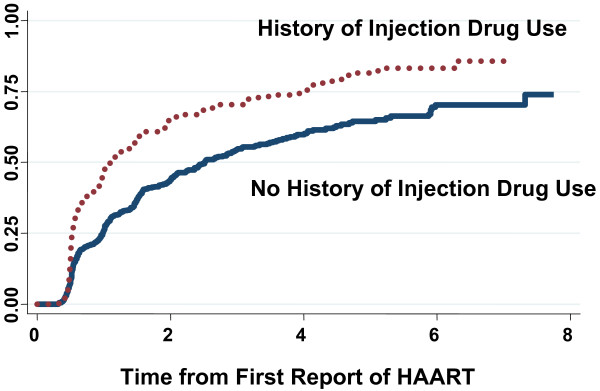
Kaplan-Meier estimates of the cumulative incidence proportion of individuals with HAART discontinuation.

For evaluating time to discontinuation, 1,588 individuals contributed 2,358 person-years with 713 events. The Kaplan-Meier estimates of the median time from first HAART report to first report of discontinuation were notably shorter for those with (1.1 years) as compared to those without (2.5 years) a history of injection drug use. Figure 3 displays the cumulative incidence proportion of HAART discontinuation for the two groups over the entire study period. These trends were supported by the results of the Cox regression analysis (Table 2). In univariate analysis, those with a history of injection drug use had a 78% higher risk of HAART discontinuation. After adjusting for pre-HAART health status and other socio-demographic variables, this elevated risk remained statistically significant (adjusted relative hazard (RH) = 1.24, 95% confidence interval (CI): 1.03–1.48). However, when restricting to data contributed after January 1999 (852 individuals contributing 382 events over 1,396 person-years), there was no increased risk seen (adj RH = 1.05, 95% CI: 0.81–1.36). Table 2 describes variables that were significant independent risk factors for discontinuation, including race/ethnicity, pre-HAART markers (CD4 and HIV RNA), prior antiretroviral therapy exposure, smoking, low income, and being unemployed.

**Table 2:** Relative hazard of HAART discontinuation (including downshifting) and HAART switching among participants initiating HAART prior to 10/1/04

	**HAART Discontinuation**			**HAART Switching**	
		
	**§Model 1:**	**§Model 2:**	**§Model 2 Restricting to data contributed after Jan 1999**	**§Model 1:**	**§Model 2:**
	**Univariate**	**Multivariate**	**Multivariate**	**Univariate**	**Multivariate**
	
**History of IDU**	**1.78 (1.53–2.06)**	**1.24 (1.03–1.48)**	1.05 (0.81–1.36)	0.96 (0.82–1.14)	1.09 (0.89–1.34)
**Age at HAART initiation (10-year increments)**	0.94 (0.85–1.03)	1.03 (0.93–1.15)	0.98 (0.84–1.15)	0.94 (0.84–1.04)	1.02 (0.91–1.14)
**Race‡**					
**Black**	**2.41 (2.02–2.88)**	**1.84 (1.47–2.29)**	**1.87 (1.37–2.54)**	0.99 (0.84–1.17)	1.19 (0.96–1.48)
**Hispanic**	**2.33 (1.95–2.92)**	**1.96 (1.50–2.57)**	**1.74 (1.15–2.65)**	**1.35 (1.09–1.68)**	**1.44 (1.10–1.87)**
**Other**	**2.86 (1.69–4.85)**	**2.39 (1.36–4.20)**	**2.64 (1.12–6.23)**	1.18 (0.61–2.30)	1.20 (0.58–2.46)
**Male**	**0.51 (0.43–0.60)**	0.85 (0.69–1.05)	1.01 (0.76–1.34)	1.00 (0.86–1.16)	1.17 (0.94–1.45)
**Nadir pre-HAART CD4+ count**	**1.23 (1.17–1.30)**	**1.39 (1.31–1.48)**	**1.48 (1.36–1.60)**	**1.18 (1.12–1.24)**	**1.28 (1.21–1.36)**
**Peak pre-HAART HIV RNA**	**1.28 (1.16–1.40)**	**1.34 (1.21–1.48)**	**1.34 (1.17–1.54)**	**1.27 (1.15–1.39)**	**1.32 (1.19–1.47)**
**Pre-HAART AIDS diagnosis**	**1.26 (1.09–1.47)**	0.96 (0.81–1.14)	0.88 (0.70–1.12)	1.17 (0.99–1.36)	1.08 (0.90–1.29)
**ART-naïve prior to HAART**	**0.77 (0.64–0.93)**	0.85 (0.70–1.05)	**0.70 (0.53–0.92)**	**0.63 (0.51–0.77)**	**0.66 (0.53–0.83)**
Alcohol use**	1.07 (0.90–1.26)	1.07 (0.89–1.28)	1.13 (0.89–1.44)	**0.79 (0.66–0.95)**	**0.81 (0.66–0.99)**
Smoking**	**1.85 (1.59–2.15)**	**1.47 (1.20–1.71)**	**1.39 (1.05–1.84)**	0.96 (0.82–1.12)	1.09 (0.91–1.30)
Employed**	**0.49 (0.42–0.58)**	**0.81 (0.67–0.98)**	0.84 (0.64–1.09)	0.80 (0.68–0.93)	0.87 (0.72–1.05)
Legal Income <$10,000**	**2.33 (2.00–2.72)**	**1.44 (1.20–1.78)**	**1.39 (1.05–1.84)**	**1.21 (1.04–1.41)**	1.18 (0.97–1.44)

§ All models adjusted for calendar time

‡ Reference category = White

**Fixed at pre-HAART baseline

In separate analyses, we examined the impact of current and former injection drug use (the latter including those individuals with a baseline history of injection drug use but no current active use). The results from these analyses were similar to those described above: for the entire dataset, there was an elevated risk of discontinuation among those reporting current injection drug use (adjusted RH = 1.65, 95% CI: 1.23–2.22) but not former users (adjusted RH = 1.16, 95% CIL 0.96–1.41) as compared with those never reporting use. These effects were attenuated when the data were restricted to 1999 and later (current users adjusted RH = 1.32, 95% CI: 0.90–1.94; former users adjusted RH = 1.00, 95% CI: 0.77–1.31).

For evaluating time to HAART regimen switch, 1,211 individuals contributed 1,931 person-years of continuous HAART use with 675 switch events. No differences in IDU were apparent in the median time to HAART switch among consistent HAART users (1.5 years for both). This was supported by the results of the Cox regression analysis (Table 2), where both univariate and multivariate analysis indicated a null association with history of injection drug use. Time-updated analysis of current injection drug use showed similar patterns: in comparison to those never reporting injection drug use, there were no significant increased risk of HAART switch among current users (adjusted RH = 1.03, 95% CI: 0.84, 1.28) or former users (adjusted RH = 1.12, 95% CI: 0.74–1.69).

## Discussion

The importance of maintaining patients on consistent, long-term HAART has been established as an effective means of suppressing HIV RNA replication and restoring immune function [1]. The initial HAART regimen is especially critical in maintaining a positive prognosis by delaying onset of clinical AIDS and allowing for future regimen options [1,24]. When HIV develops resistance to a drug, it often develops cross-resistance with several drugs in the same class, severely limiting future treatment alternatives [25]. However, despite these concerns, many patients may need to modify therapy in order to find a regimen that is more suitable for them, whether to avoid side effects or increase convenience, or due to virologic failure [12].

Such treatment issues are of particular concern among HIV-infected injection drug users [26]. Findings from an earlier study by Chen et al [14] found that a history of injection drug use was independently associated with the probability of switching or discontinuing a HAART regimen. In this study, we observed that time from HAART initiation to first regimen modification was significantly shorter among injection drug users. However, after adjusting for potential confounders and examining data in the era of more advanced HAART regimens, we observed that the hazard of HAART modification was attenuated and not significantly different between those with and without a history of injection drug use.

These results suggest that other factors may be leading to the increased risk of HAART modifications in the ALIVE study and among IDU populations in general. We recognize that a history of IDU in itself may be both confounded and a consequence of a variety of factors (e.g. incarceration, history of physical abuse, etc). Income level, employment, smoking and alcohol use were shown to confound the relationship (e.g. the effect of IDU was attenuated in Model 2 as compared to Model 1) but adjusting for them did not completely eliminate the association between IDU history and HAART modification. We did identify several factors that proved to be predictive of HAART modification, the strongest and most consistent were measures of HIV disease progression (a higher pre-HAART peak HIV RNA level, pre-HAART antiretroviral experience) but also and minority race/ethnicity status. These findings are consistent with most prior studies that have investigated HAART regimen modifications [11,12,16,17], and suggests that these modifications may occur due to failure of the previous regimen, and may also be linked to access to care. Previous work by Silverberg *et al *[27] working with the US military cohort (which has racial/ethnic diversity but universal access to health care) has not demonstrated any differences in HAART effectiveness by race, thus suggesting that socio-behavioral factors (e.g. access or quality of care, clinical depression, therapy adherence) may be more likely determinants of HAART success. Unfortunately, these were not uniformly measured in the three cohorts and therefore could not be assessed as potential confounders in our analyses.

The main limitation of this study is that all data on antiretroviral use and HAART modification were based on self-report. Therefore, it is possible that participants may have misreported or underreported what medications they were using. However, data on antiretroviral use were obtained using photo-medication study aids to facilitate recall of drug usage and both the common and brand names were used in the interview. Additionally, in an unpublished analysis performed on a convenience sample subset of the MACS participants, in which the medical records were available and abstracted to compare with the self-reported medication data, it was found that 95% of the drug records were in concordance with the self-reported data (L. Jacobson, personal communication). However, this sub-study was performed only in the MACS and it is possible that some of the differences seen between the MACS and ALIVE are due to a higher level of misreport or lack of recall in the ALIVE study. If true, this would be an indication of the necessity of increased patient education among IDUs regarding the medications they are using with emphasis on the importance of adherence.

The lack of data on viral drug resistance profiles and co-morbidities among the participants in each cohort is an additional limitation of this study. Drug resistance and co-morbidities are an important potential reason for HAART therapy modification that we were not able to investigate in this study. A strength of the current study, however, is its prospective design, allowing for determination of exposure variables prior to the outcome (e.g. HAART modifications) using standardized protocols implemented as part of three long-running *interval *cohort studies [28]. Additionally, the common design of all three parent studies and data collection instruments facilitated comparisons between the cohorts using compatible covariates and a common algorithm for the determination of HAART modifications.

In conclusion, we have demonstrated that in more recent years there is no impact of reported use of injection drugs on HAART modification. These data imply efforts to simplify and increase potency of antiretroviral regimens have been successful at improving HAART maintenance among a group at high risk for failure. However, the persistent association of race/ethnicity and low income with HAART discontinuation provides important direction and motivation for improving methods for HAART utilization among these high-risk groups.

## Competing interests

The author(s) declare that they have no competing interests.

## Authors' contributions

All authors have: 1) made substantial contributions to conception and design, acquisition of data, and analysis and interpretation of data; 2) have been involved in drafting the manuscript; and 3) have read and given approval of the final version of the manuscript.
